# Lactate/pyruvate transporter MCT-1 is a direct Wnt target that confers sensitivity to 3-bromopyruvate in colon cancer

**DOI:** 10.1186/s40170-016-0159-3

**Published:** 2016-10-03

**Authors:** Stephanie Sprowl-Tanio, Amber N. Habowski, Kira T. Pate, Miriam M. McQuade, Kehui Wang, Robert A. Edwards, Felix Grun, Yung Lyou, Marian L. Waterman

**Affiliations:** 1 Department of Microbiology and Molecular Genetics, University of California, Irvine, Irvine, CA USA; 2 Department of Pathology and Laboratory Medicine, University of California, Irvine, Irvine, CA USA; 3 Department of Developmental and Cell Biology, University of California, Irvine, Irvine, CA USA

**Keywords:** Monocarboxylate transporter 1 (MCT-1), *SLC16A1*, Wnt signaling, Colon cancer, Metabolism, 3-Bromopyruvate, XAV939

## Abstract

**Background:**

There is increasing evidence that oncogenic Wnt signaling directs metabolic reprogramming of cancer cells to favor aerobic glycolysis or Warburg metabolism. In colon cancer, this reprogramming is due to direct regulation of pyruvate dehydrogenase kinase 1 (*PDK1*) gene transcription. Additional metabolism genes are sensitive to Wnt signaling and exhibit correlative expression with PDK1. Whether these genes are also regulated at the transcriptional level, and therefore a part of a core metabolic gene program targeted by oncogenic WNT signaling, is not known.

**Results:**

Here, we identify monocarboxylate transporter 1 (MCT-1; encoded by *SLC16A1*) as a direct target gene supporting Wnt-driven Warburg metabolism. We identify and validate Wnt response elements (WREs) in the proximal *SLC16A1* promoter and show that they mediate sensitivity to Wnt inhibition via dominant-negative LEF-1 (dnLEF-1) expression and the small molecule Wnt inhibitor XAV939. We also show that WREs function in an independent and additive manner with c-Myc, the only other known oncogenic regulator of *SLC16A1* transcription. MCT-1 can export lactate, the byproduct of Warburg metabolism, and it is the essential transporter of pyruvate as well as a glycolysis-targeting cancer drug, 3-bromopyruvate (3-BP). Using sulforhodamine B (SRB) assays to follow cell proliferation, we tested a panel of colon cancer cell lines for sensitivity to 3-BP. We observe that all cell lines are highly sensitive and that reduction of Wnt signaling by XAV939 treatment does not synergize with 3-BP, but instead is protective and promotes rapid recovery.

**Conclusions:**

We conclude that MCT-1 is part of a core Wnt signaling gene program for glycolysis in colon cancer and that modulation of this program could play an important role in shaping sensitivity to drugs that target cancer metabolism.

**Electronic supplementary material:**

The online version of this article (doi:10.1186/s40170-016-0159-3) contains supplementary material, which is available to authorized users.

## Background

Canonical Wnt signaling regulates the fate and activities of cells through the actions of β-catenin, a nuclear-localizing mediator that can activate the transcription of Wnt target genes important in cell growth and proliferation. A chronic increase in the cellular levels of β-catenin can occur through oncogenic activation of the Wnt signal transduction pathway, a condition that leads to aberrant and elevated expression of Wnt target genes. Constitutive Wnt target gene expression is an abnormal condition that can transform cells and cause cancer, including colon cancer, a disease defined by epithelial cell transformation within the intestine. Colon cancer most commonly derives from chronic activation of the canonical Wnt signaling pathway through mutations of components in the destruction complex, a multi-subunit regulator in the cytoplasm that degrades β-catenin to maintain appropriate, physiological levels [1–4]. Nuclear-localized β-catenin activates target gene expression via direct binding to LEF/TCF transcription factors, a family of four DNA binding proteins that occupy distinct gene targets (i.e., gene programs) and direct specific phenotypes and functions of cells. Gene programs identified to be altered by oncogenic Wnt signaling include cell cycle progression, epithelial-mesenchymal transition (EMT), angiogenesis, migration, cell survival, and most recently discovered by our group, metabolism [5–8].

Many groups have used overexpression of dominant-negative isoforms of LEF/TCF transcription factors (dnLEF/TCFs) in multiple contexts and model systems to identify Wnt target genes [5, 6, 9, 10]. These shorter forms retain the capabilities of full length LEF/TCFs to occupy Wnt response elements (WREs) throughout the genome, but they lack the ability to recruit β-catenin. Interference by these dominant-negative isoforms represses target gene transcription, and thus, genome-wide expression analysis of downregulated transcription can reveal candidate target genes and the gene programs with which they are associated. We used this type of analysis in colon cancer cells to discover that Wnt signaling promotes tumor cell preferences for aerobic glycolysis/Warburg metabolism, with the Wnt target gene pyruvate dehydrogenase kinase 1 (PDK1) playing a significant role in this metabolic fate [8]. In that study, we also observed additional metabolism-linked genes to be sensitive to dnLEF/TCF expression, suggesting that Wnt signaling coordinately regulates PDK1 within a larger gene program. One of the additional genes affected was monocarboxylate transporter 1 (*SLC16A1*, encoding the protein MCT-1), a known lactate transporter observed to be upregulated in many cancers [11, 12]. qRT-PCR analysis of xenograft tumors from a colon cancer cell line showed MCT-1 downregulation in the presence of dnLEF/TCFs, and ChIP-seq ENCODE data shows TCF-4 occupancy of *SLC16A1* in HCT116 colon cancer cells [8]. These preliminary findings strongly implicate MCT-1 as a direct Wnt target gene that might be coordinately regulated with PDK1. Here, we investigate this possibility and show that MCT-1/*SLC16A1* is a direct target gene of β-catenin-LEF/TCF complexes in colon cancer cells.

MCT-1 is one of 14 members of the *SLC16* family of transporters [13]. While the functions of many MCT family members remain uncharacterized, MCT-1 through MCT-4 is confirmed proton-linked monocarboxylic acid transporters [14]. These four family members have been shown to transport monocarboxylates including acetoacetate, β-hydroxybutyrate, short chain fatty acids, pyruvate, and lactate. In a normal setting, MCTs are necessary for lactate efflux from highly glycolytic/hypoxic muscle fibers during exercise, and also reabsorption or uptake of monocarboxylates from the gut, liver, and kidney for gluconeogenesis or lipogenesis—activities tightly linked to aerobic and anaerobic glycolysis [14]. MCT-1 has a reasonably strong affinity for lactate compared to the other MCTs (K_m_ of 2.5–4.5 mM, compared to MCT-2 K_m_ = 0.7 mM; MCT-3 K_m_ = 6 mM; MCT-4 K_m_ = 17–34 mM), and it is broadly expressed, while other MCT family members are localized to specific regions of the body at varying levels of expression [13, 15].

While increased expression of MCT-1 in response to the physiological stresses of exercise and physical stimulation has been well defined, the molecular mechanisms that govern its expression are still poorly understood. At the transcriptional level, the *SLC16A1* promoter contains nuclear factor of activated T-cells (NFAT)-binding sequences [14], but the significance of these elements is unknown. In rat skeletal muscle tissues, PGCα (a transcriptional co-activator linked to regulation of genes involved in energy metabolism) has been associated with MCT-1 upregulation in response to muscle activity [16]. However, no follow-up studies have been conducted to determine whether the *SLC16A1* promoter is subject to direct activation. The ribonucleotide metabolite and AMP-activated protein kinase (AMPK) activator, 5-aminoimidazole-4-carboxamide-1-β-d-ribonucleoside (AICAR), has been shown to upregulate or downregulate *SLC16A1* promoter activity depending on the study and tissue context [17]. Likewise, butyrate, another metabolite and energy source for the colon epithelium has been identified to enhance transcription and transcript stability of *SLC16A1* mRNA [18], but the mechanisms and responsive genomic regions behind these effects are not known. Finally, hypoxia was shown to upregulate MCT-1 in human adipocytes [19], but this is a singular example. In most tissues and cell lines studied, MCT-1 expression is not affected by hypoxia [20]. Instead, MCT-4 is considered to be the main transcriptional responder to hypoxia as multiple, high affinity HIF response elements (HREs) have been identified in its promoter and hypoxic expression has been demonstrated in many tissues [20].

The observation that MCT-1 expression is increased in cancer has led to studies focused on its regulation in cancer cells. For example, the tumor suppressor p53 directly binds to the MCT-1 promoter for transcription repression, and therefore, the loss of p53 in cancer cells enables MCT-1 mRNA production [21]. c-Myc also directly regulates MCT-1 transcription, especially in cancer cells where high levels of c-Myc drive metabolic pathways [22]. A common theme among cancer cells is the use of elevated MCT-1 expression to support the glycolytic preference of cells via its ability to export lactate. This export minimizes the cellular stresses from acid buildup and maintains proper intracellular pH, activities crucial to cancer cell survival [23]. Alternatively, a recent study found that MCT-1 primarily exports pyruvate, where co-expressed MCT-4 plays the dominant role in exporting lactate [24]. This function appears to promote glycolysis as inhibition of MCT-1 transporter activity or downregulation of its protein levels leads to increased oxidative phosphorylation and decreased proliferation [24]. Taken together, no matter the precise actions of its transporter functions, the use of MCT-1-specific inhibitors has shown this transporter to be a key player in cancer cell metabolism, survival, and proliferation, making it a potentially important candidate target in glycolytic cancer cells [25].

Recent findings highlight how MCT-1 overexpression may be an exploitable feature for cancer therapy. Birsoy et al. have shown that breast cancer cells expressing MCT-1 are sensitive to 3-bromopyruvate (3-BP), a molecule that can have anti-proliferative effects by targeting glycolytic enzymes and other metabolic pathways [26]. Like its parent molecule pyruvate, 3-BP must be transported across the plasma membrane. Birsoy et al. used genome-wide screening to discover that 3-BP is imported into cells strictly through MCT-1 and no other transporter or alternative pathway. Whether this makes MCT-1 expression the single most important biomarker for determining tumor sensitivity to 3-BP is not yet known, as its precise mode of action has not been defined and only breast cancer cells were used in the study. Nevertheless, there are several case reports documenting the use of this compound in cancer patients, underscoring the importance of understanding how *SLC16A1* gene expression is regulated [27, 28]. Here, we show that MCT-1/*SLC16A1* is a direct Wnt target gene coordinately regulated with other genes that promote glycolysis in colon cancer cells. We define a region in the upstream promoter with at least two WREs and show that the endogenous gene is sensitive to dnLEF/TCF inhibition in multiple colon cancer cell lines. We show that transcriptional regulation by β-catenin/LEF/TCFs is separate and additive with c-Myc action. We demonstrate that colon cancer cells are sensitive to 3-BP and that the sensitivity tracks partially, but not completely, with the strength of oncogenic Wnt signaling. Finally, we show that Wnt signaling inhibitors do not synergize with 3-BP to suppress proliferation, but instead interfere with the anti-proliferative effects of 3-BP and provide a resistance mechanism for colon cancer cells.

## Results and Discussion

### MCT-1 is regulated by Wnt signaling

Our recent discovery showing that Wnt signaling directs colon cancer cells to utilize glycolysis specifically focused on Wnt regulation of target gene pyruvate dehydrogenase kinase 1 (*PDK1*), a mitochondrial kinase that suppresses pyruvate uptake by mitochondria to favor conversion to lactate in the cytoplasm. We utilized a microarray analysis of dnLEF/TCF isoform induction in the colon cancer cell line DLD-1 to reveal novel roles of Wnt signaling in colon cancer. Gene ontology analysis of the entire gene expression dataset revealed that additional metabolic genes might be coordinately regulated with *PDK1* and might contribute to the effect Wnt signaling has on tumor cell metabolism. In this study, we focus on the lactate transporter *SLC16A1*/MCT-1 because it lies downstream of *PDK1* and glycolysis to export metabolites such as lactate, and because it is the importer of 3-BP. We ask if MCT-1 is directly regulated by β-catenin/LEF/TCF complexes and if this regulation is an important consideration for cancer therapies that target metabolism.

**Fig. 1:**
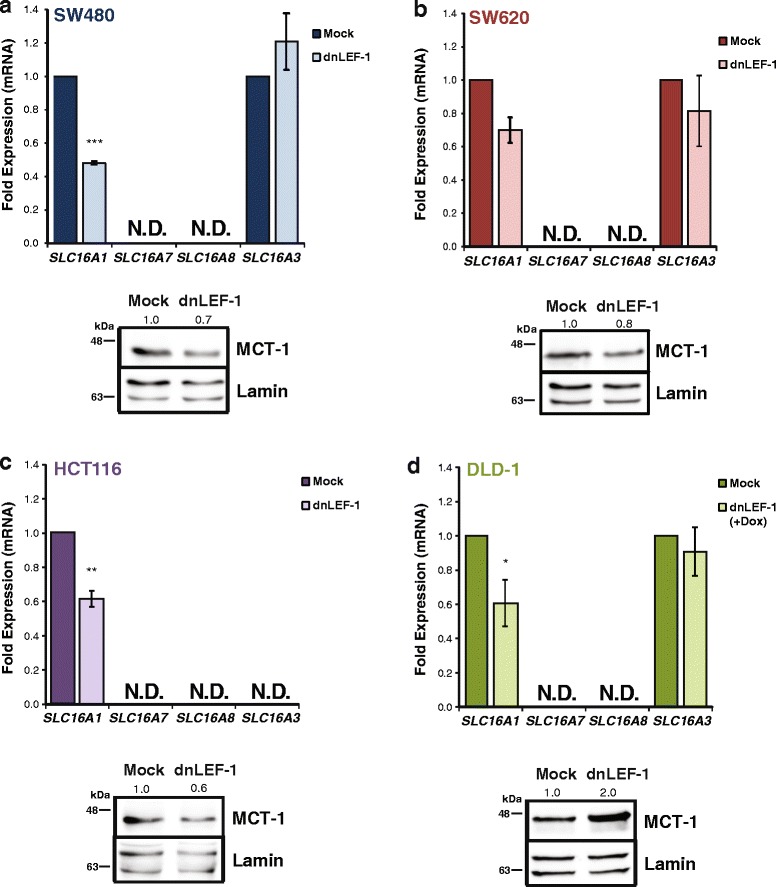
Blocking Wnt with dnLEF-1 reduces MCT-1 but not MCT-4 levels. qRT-PCR analysis was performed on RNA collected from SW480 (**a**) and SW620 (**b**) cells stably expressing dnLEF-1. Analysis was also performed for HCT116 cells (**c**) 72 h after lentiviral transduction of dnLEF-1 and DLD-1 dnLEF-1 cells (**d**) harvested 72 h after the addition of doxycycline. Graphs shown represent the average of three trials (+/− SEM). Whole cell lysates from each cell line (**a**–**d**) were harvested concurrently with RNA and were probed with the antibodies shown. (*p value <0.05; **p value < 0.01; ***p value < 0.001)

To validate the microarray results in DLD-1 cells and to expand the analysis to additional colon cancer cell lines, we used qRT-PCR to measure how *SLC16A1* mRNA levels change in response to modulation of β-catenin and LEF/TCFs. *SLC16A1* mRNA was purified from SW480 cells (Fig. 1a) and SW620 cells (Fig. 1b) that had been stably transduced with lentivirus expressing physiological levels of dnLEF-1. We found that dnLEF-1 expression reduced *SLC16A1* mRNA to 50 % (SW480) and 70 % (SW620) of parental levels, suggesting that endogenous LEF/TCF/β-catenin complexes are contributing to *SLC16A1* transcription. In a separate study in SW480 cells, shRNA-mediated reduction of β-catenin reduced *SLC16A1* mRNA levels to 35 % of control levels [29]. *SLC16A1* mRNA levels were also determined for HCT116 cells with dnLEF-1 (Fig. 1c) and doxycycline-induced dnLEF-1 DLD-1 cells for comparison to the microarray analysis (Fig. 1d). In these latter two cases, we observed a reduction of *SLC16A1* mRNA to approximately 60 % in each cell line.

Since there are four *SLC16* family members that are known to export lactate (MCT1-4), we asked whether any of these were also downregulated after inhibiting Wnt signaling. We used qRT-PCR analysis to assess the expression of the four genes (MCT-1, MCT-2, MCT-3, and MCT-4) in SW480, SW620, HCT116, and DLD-1 cells under the previously mentioned conditions. We found that both *SLC16A7/*MCT-2 and *SLC16A8*/MCT-3 were undetectable in these four cell lines. *SLC16A3*/MCT-4 mRNA was easily detected, but there were no statistically significant changes in levels upon inhibition of Wnt signaling, (albeit *SLC16A3/*MCT-4 was not detected in HCT116 cells; Fig. 1a–d). We also tested for differences at the protein level using western blot analysis. MCT-1 levels were reduced by 20–40 % for SW480, SW620, and HCT116 cells, similar to the reduction observed at the mRNA level (Fig. 1a–c). In contrast, DLD-1 cells showed a twofold increase in protein level with dnLEF-1 expression, suggesting that MCT-1 may be regulated differently in this cell line compared to the others, possibly as unique compensatory changes unfold under stable, chronic expression of dnLEF1 (Fig. 1d). We also tested for MCT-1 expression following acute interference of Wnt signaling. The small molecule inhibitor XAV939 acts by suppressing tankyrase 1/2—poly-ADP-ribosylating enzymes that de-stabilize the destruction complex via PARsylation-directed ubiquitination of axin, a key scaffolding subunit [30]. XAV939 can therefore trigger a rapid decrease in β-catenin levels via increased activity of the destruction complex. We treated each cell line with XAV939 for 24 h and used qRT-PCR to quantitate mRNA levels (Additional file 1: Figure S1). Cell lines were treated with XAV939 for 24 and 72 h for western blot analysis to quantitate β-catenin and MCT-1 protein levels, respectively (Additional file 1: Figure S1). We observed that MCT-1 expression was significantly reduced in all four cell lines.

**Fig. 2:**
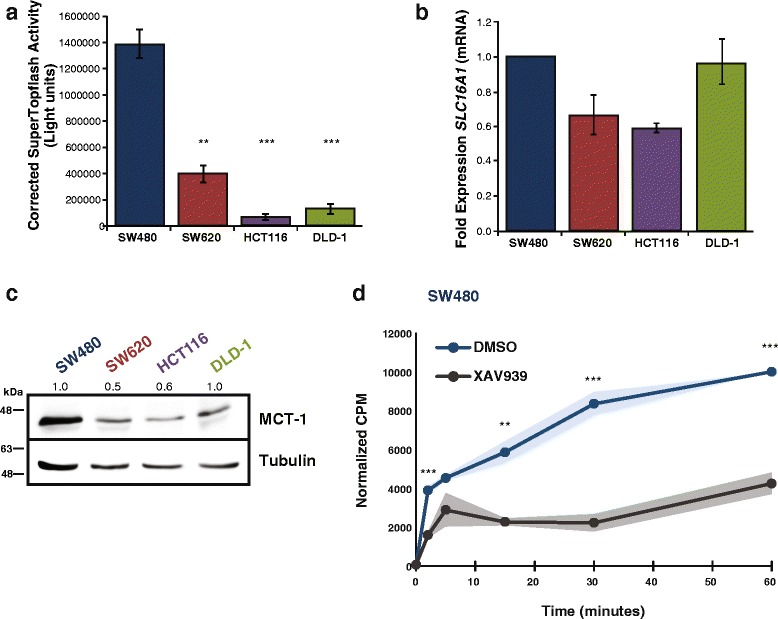
Wnt signaling correlates with MCT-1 levels and activity in colon cancer cells. **a** Luciferase reporter activity in parental SW480, SW620, HCT116, and DLD-1 cells shows varying levels of Wnt signaling based on SuperTopflash activity. Graph represents the average of three trials (+/− SEM). (*p value < 0.05; **p value < 0.01; ***p value < 0.001). **b** qRT-PCR analysis was performed on RNA collected from parental SW480, SW620, HCT116, and DLD-1 cells. Graph represents the average of three trials with fold change over SW480 cells (+/− SD). **c** Whole cell lysates from each cell line were collected and probed with the antibodies shown. **d** Radiolabeled ^14^C pyruvate uptake assay on SW480 cells treated with Wnt signaling inhibitor XAV939 (72 h) or vehicle (DMSO). Graph represents average of *n* = 4 with the shaded area showing +/− SEM (*p value < 0.05; **p value < 0.01; ***p value < 0.001)

We next asked whether MCT-1 levels correlated with the level of Wnt signaling. To test this, we transfected SW480, SW620, HCT116, and DLD-1 cells with SuperTopflash, a Wnt signaling luciferase reporter plasmid regulated by an array of seven WREs and a minimal promoter [31]. Each cell line exhibited varying levels of Wnt signaling with SW480 cells showing the highest level of activity by far. SW620 cells had 35-fold less activity in comparison, and HCT116 and DLD-1 cells exhibited the lowest levels (Fig. 2a). We hypothesized that if *SLC16A1* is a direct target of Wnt signaling, the relative level of mRNA in each of the cell lines would correlate with the activity level of the SuperTopflash reporter. We performed qRT-PCR analysis of *SLC16A1* mRNA for each cell line and normalized the results to SW480 levels (Fig. 2b). We observed that SW620 and HCT116 cells had lower *SLC16A1* mRNA transcripts compared to the “Wnt^**Hi**”^ SW480 cells. We also compared protein levels using western blot analysis, normalizing protein level to SW480 cells for comparison (Fig. 2c). MCT-1 protein levels were lower for SW620 and HCT116 cells (50–60 %), reflective of the relatively lower *SLC16A1* mRNA levels in these cells. DLD-1 cells differed from the correlation in that even though the SuperTopflash activity was one of the lowest, the mRNA and protein levels were similar to Wnt^**Hi**^ SW480 cells. Since MCT-1 is also a target of c-Myc, we used XAV939 treatment to assess the contribution of β-catenin regulation to MCT-1-specific activities. We developed a ^14^C-pyruvate uptake assay since this capability distinguishes activity unique to MCT-1 compared to the co-expressed MCT-4 transporter. We used our standard XAV939 concentration that partially lowers β-catenin protein levels so as not to be lethal or affect c-Myc expression (data not shown), and observed a decrease in the rate of pyruvate uptake in XAV939-treated SW480 colon cancer cells (Fig. 2d; ~2000 cpm/min reduced to ~800 cpm/min). The 60 % decrease in initial rate aligns very well with the 50 % decrease in MCT-1 protein levels. We also evaluated intracellular and extracellular lactate and oxidized glutathione content in XAV939-treated, as well as in dnLEF-1-expressing SW480 cells (Additional file 2: Figure S2). We observed that extracellular (secreted) lactate levels were significantly reduced in XAV939 and dnLEF-1 conditions, but intracellular lactate concentrations were similar between control and treated samples. The total amount of lactate in the media (1 μmole) was approximately 50-fold greater than that in the cells (20 nmoles). These data show that lactate production (glycolysis) is reduced when Wnt signaling is inhibited (a finding that we have previously reported), but that the ability of the cells to efficiently export lactate is not affected even though MCT-1 levels are reduced. We attribute this to the fact that MCT-4 can compensate for lactate transport, an activity reported by other groups [25, 32]. Since others have shown that disruption of MCT-1 function can lead to multiple metabolic changes including decreases in glutathione (GSH) and the emergence of reactive oxygen species (ROS) [22], we evaluated these two compounds. While we did not observe significant changes in GSH content in cells (data not shown), we did observe modest increases in ROS, albeit not quite statistically significant (Additional file 2: Figure S2b). Overall, these results suggest that MCT-1/*SLC16A1* is regulated by Wnt/β-catenin signaling in colon cancer cells, and with DLD-1 cells as the one exception, MCT-1/*SLC16A1* RNA, protein and activity levels correlate with the relative levels of canonical Wnt signaling.

### MCT-1 is a direct target of Wnt signaling

**Fig. 3:**
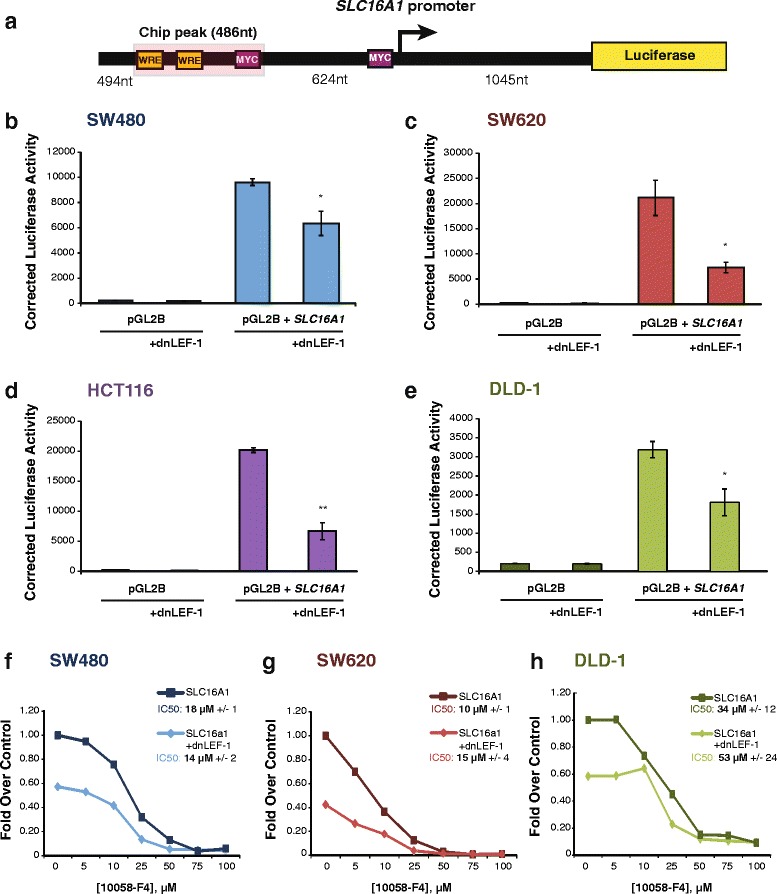
Wnt directly targets the MCT-1/*SLC16A1* promoter for regulation. A schematic (**a**) depicts a region of the endogenous *SLC16A1* promoter (−1604, +1045) that was subcloned into a luciferase reporter plasmid. One regulatory region located approximately 624 nt upstream from the *SLC16A1* transcription start site (+1) is occupied by dnTCF-1 and contains two putative Wnt response elements (highlighted in *yellow*). Previously identified c-Myc binding sites are also represented (in *purple*). Transient transfection analysis of three independent experiments in SW480 (**b**), SW620 (**c**), HCT116 (**d**), and DLD1 (**e**) cells shows that the endogenous promoter fragment increases transcription, and that co-expression of dnLEF-1 reduces activity of this promoter construct. Graphs shown represent the average of three trials (+/− SEM; *p value < 0.05; **p value < 0.01; ***p value < 0.001). Luciferase reporter activity in SW480 (**f**), SW620 (**g**), and DLD1 (**h**) cells shows that treatment with the Wnt inhibitor XAV939 (10 μM) and increasing concentrations of c-Myc inhibitor 10058-F4 decrease transcription of the SLC16A1 promoter additively, but not synergistically. A representative graph is shown of three replicates, with calculation of the IC_50_ and SEM from all three replicates for each cell line in the legend

To ask whether Wnt/β-catenin regulation of MCT-1/*SLC16A1* expression is direct or indirect, we mined a previously performed genome-wide ChIP-seq data set of dnTCF-1 binding in DLD-1 cells and discovered that TCF-1 binds to a region in the *SLC16A1* locus (Fig. 3a) [33]. This region (486 nucleotides; “ChIP peak”) of occupancy contains two putative WREs (sequence, Additional file 3: Figure S3). We also note that Watanabe et al. identified this same region as a site of β-catenin occupancy in SW480 cells [29]. To test whether the promoter region confers active transcription regulation in colon cancer cells, we subcloned a fragment of the genomic locus encompassing the ChIP peak and the transcription start site next to the luciferase open reading frame in the plasmid pGL2b. Using “empty” pGL2b plasmid activity as a negative control and SuperTopflash activity as a positive control, the transient transfection assays showed that the promoter fragment increased reporter activity over empty vector in each of the four surveyed lines (SW480 44-fold; SW620 98-fold; HCT116 83-fold; DLD-1 16-fold), and that it was specifically sensitive to downregulation when dnLEF-1 was co-expressed (Fig. 3b–e, Additional file 3: Figure S3).

Two c-Myc binding sites have been previously identified within the promoter region of the gene (−624 to the transcription start site) and shown to regulate *SLC16A1* transcription [22]. Since c-Myc is a well-established Wnt target gene, we asked whether c-Myc and LEF/TCF-β-catenin complexes synergize mechanistically to activate RNA polymerase II transcription of the *SLC16A1* locus. To examine this, we transfected the *SLC16A1* reporter into SW480, SW620, and DLD-1 cells and then treated the cultures with an increasing dose of the small molecule c-Myc inhibitor 10058-F4, which prevents c-Myc-Max interaction. The inhibitor reduced promoter activity in all lines at similar IC_50_, with DLD-1 cultures showing a modest level of decrease in sensitivity (Fig. 3f–h). A parallel set of cultures in which dnLEF1 was expressed to partially lower Wnt signaling were treated with the same dose response regimen (Fig. 3f–h). While the combination of Wnt and c-Myc inhibition had clear, additive effects on transcription, there was no significant difference in the IC_50_ for 10058-F4 alone compared to its effects in the presence of dnLEF-1. This result suggests that c-Myc:Max and LEF/TCF-β-catenin actions influence the same or similar steps of transcription.

**Fig. 4:**
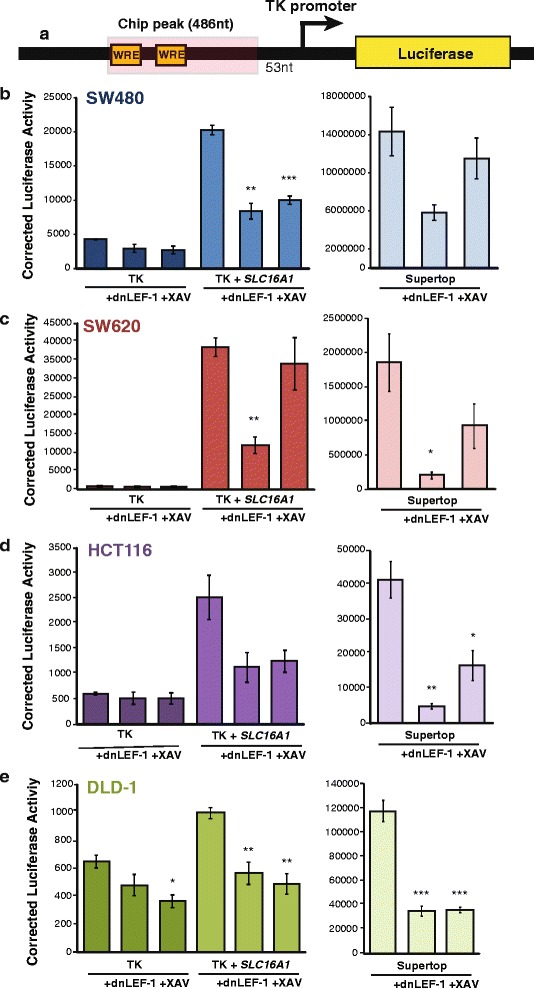
Identification of a Wnt responsive region in the *SLC16A1* promoter region. A schematic (**a**) representing the ChIP peak region (486 nt) occupied by dnTCF-1, which was subcloned 5′ of the heterologous thymidine kinase (TK) core promoter and luciferase open reading frame. Luciferase reporter activity in SW480 (**b**), SW620 (**c**), HCT116 (**d**), and DLD1 (**e**) cells shows that the ChIP peak region confers elevated transcription activity to the heterologous TK promoter. The expression of transfected dnLEF-1, or treatment with the Wnt inhibitor XAV939 (10 μM) reduces the regulatory activity of these fragments. Graphs shown represent the average of three independent replicates (+/− SEM) (*p value < 0.05; **p value < 0.01; ***p value < 0.001)

To confirm that the putative Wnt response elements confer transcription regulation to a heterologous promoter in colon cancer cells, we subcloned the ChIP peak next to the thymidine kinase (TK) promoter and luciferase open reading frame (Fig. 4a). Luciferase activity assays were performed in the presence of dnLEF-1 or Wnt inhibitor XAV939, showing that the fragment increased promoter activity in SW480, SW620, HCT116, and DLD-1 colon cancer cells (Fig. 4b–e). The induction of dnLEF-1 expression reduced luciferase expression to near baseline in all the cell lines, and treatment with Wnt inhibitor XAV939 also repressed reporter expression, but with more variability (Fig. 4b–e). The ChIP peak fragment exhibited more activity in SW480 cells and SW620 cells compared to HCT116 cells and DLD-1 cells, tracking better with the activity profile of the SuperTopflash reporter (Fig. 2a). These results demonstrate that *SLC16A1*/MCT-1 is a direct Wnt target gene and that regulation occurs through sites within the promoter locus. Therefore, MCT-1 is part of a metabolic/glycolytic gene program directly targeted by Wnt signaling.

### Wnt signaling inhibition increases colon cancer cell resistance to 3-bromopyruvate

**Fig. 5:**
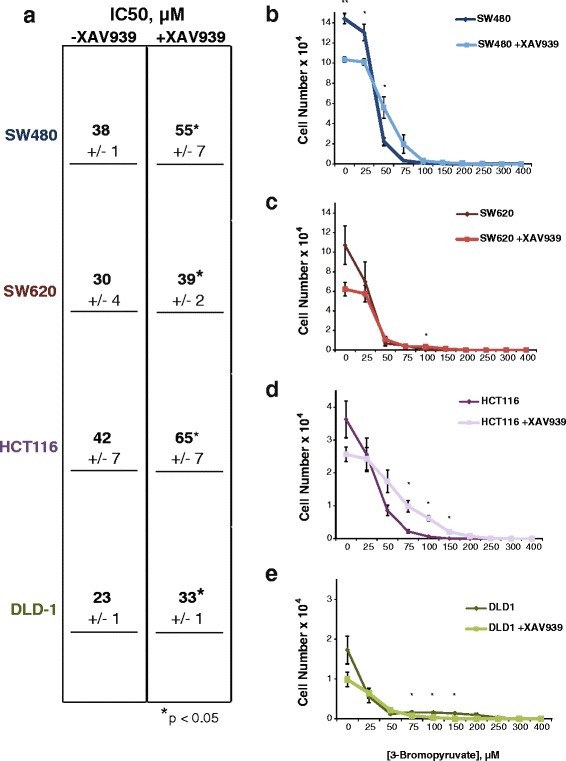
XAV939 affects colon cancer cell sensitivity to 3-bromopyruvate. **a** Table of IC_50_ values for 3-bromopyruvate in SW480, SW620, HCT116, and DLD-1 cells. Cell cultures were pre-treated with or without XAV939 (10 μM) for 48 h prior to the addition of 3-BP. SW480 (**b**), SW620 (**c**), HCT116 (**d**), and DLD1 (**e**) cells pre-treated with the Wnt inhibitor XAV939 (10 μM) reduces survival compared to without 3-bromopyruvate treatment, except at high concentrations. Graphs shown represent the average of three independent replicates with error bars depicting the SEM (*p value < 0.05; **p value < 0.01; ***p value < 0.001)

The importance of MCT-1 to cancer cell survival has been well characterized in other cancers [25, 34–36], with a recent study identifying a potential glycolysis inhibitor that targets cells via import through MCT-1 [26]. In fact, Birsoy et al. used a genome wide siRNA knockdown screen to discover that MCT-1 and Basigin (the transmembrane glycoprotein responsible for anchoring MCT-1 to the cell surface [37, 38]) are uniquely and sufficiently capable of importing the toxic molecule 3-BP into breast cancer cells. Breast cancer cell lines expressing high levels of MCT-1 were exquisitely sensitive to treatment with 3-BP, while cell lines that did not express MCT-1 were resistant and survived even in the presence of the molecule. Furthermore, knockdown or overexpression of MCT-1 in breast cancer cell lines enhanced or prohibited survival, respectively. Since that study focused exclusively on breast cancer cell lines, we asked what effect 3-BP would have on colon cancer cells. Given that Birsoy et al. showed a direct correlation between MCT-1 levels and sensitivity to 3-BP, we asked whether there is a correlation between the level of Wnt signaling and 3-BP sensitivity in colon cancer cells. We also performed the 3-BP dose-response analysis in the presence and absence of Wnt signaling inhibitors, which addressed a second question, namely, whether reduction of β-catenin levels would enhance any negative effect of 3-BP on cell growth or whether it would produce complex protective effects by lowering MCT-1 expression. We first subjected cells to 48 h of vehicle or XAV939 to lower MCT-1 protein levels, and then followed that treatment with 96 h of increasing doses of 3-BP (Fig. 5). We observed that colon cancer cells are as sensitive, if not more so, than breast cancer cell lines, and that while XAV939 treatment provided additional inhibitory effects on growth in the presence of low concentrations of 3-BP, it appeared to be somewhat protective at higher doses (Fig. 5b–e). This result was further supported by IC_50_ analyses which showed that XAV939 caused statistically significant increases in the 3-BP IC_50_ for all of the cell lines, with the most notable differences evident in SW480 and HCT116 cells. This data led us to ask whether the partial protection provided by XAV939 would be evident in the cultures even after all the drugs were removed, affecting the survival and recovery of colon cancer cells from toxic, high doses of 3-BP.

**Fig. 6:**
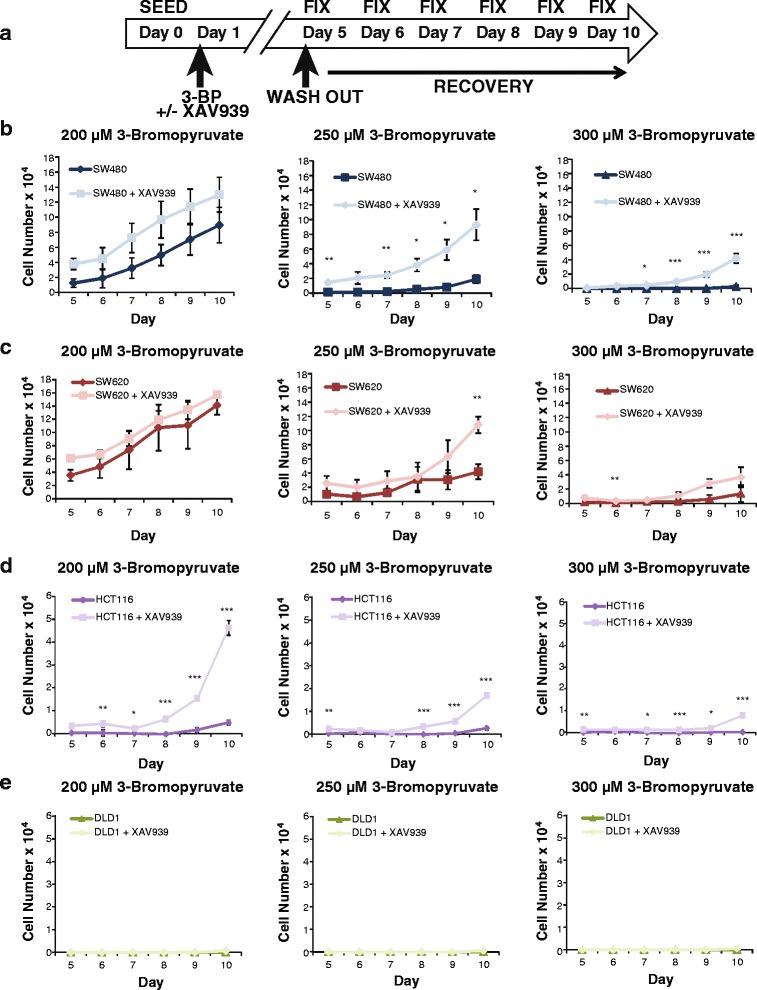
XAV939 promotes colon cancer cell recovery from 3-bromopyruvate. **a** Experimental workflow for colorimetric-based survival curve assay. Individual cell lines were seeded on day 0 and treated after 24 h (day 1) with 200, 250, or 300 μM 3-bromopyruvate +/− Wnt signaling inhibitor XAV939 (10 μM) until day 5. On day 5 treatments were “washed out” and replaced with control medium. Cells were fixed on days 5–10 post seeding to observe recovery over time. SW480 (**b**), HCT116 (**d**) cells treated with the Wnt signaling inhibitor XAV939 (10 μM) survived and recovered compared to without treatment of 3-bromopyruvate. SW620 (**c**) cells recovered similarly +/− XAV939 treatment, and DLD1 cells (**e**) did not recover. Graphs shown represent the average of three independent replicates (+/− SEM) (*p value < 0.05; **p value < 0.01; ***p value < 0.001)

To test this notion of survival, the four surveyed cell lines were treated with 200, 250, or 300 3-BP—concentrations of drug that were seven- to tenfold above the IC_50_. These treatments were followed by a “wash out” and recovery period for 5 days (Fig. 6a). In SW480 cells treated with 200 μM 3-BP, there was no significant difference in the ability of the cells to recover in the absence or presence of XAV939. However, at 250 μM, the cells survived significantly better if they had been treated with XAV939, and at 300 μM 3-BP, Wnt signaling inhibition allowed the cells to recover where untreated cultures did not recover at all (Fig. 6b). Similarly in SW620 cultures, XAV939 treatment increased survival and recovery at 250 μM 3-BP, whereas the 300 μM condition had a modest recovery (Fig. 6c). Wnt signaling-inhibited cells survived and recovered better at every experimental concentration of 3-BP in HCT116 cells (Fig. 6d), but DLD-1 cells did not recover at all. The lack of DLD-1 cell recovery was unsurprising, due to this cell line having the lowest IC_50_ and greatest sensitivity to 3-BP (Figs. 5e and 6e). These data demonstrate that even though colon cancer cells are sensitive to 3-BP, co-treatment with Wnt signaling inhibitor XAV939, which lowers MCT-1 expression, allows cells to resist the toxic effects of 3-BP leading to increased survival and enhanced recovery from a minor fraction of surviving cells. These results reveal an important drug combination that should be avoided when trying to treat cancer cells with aberrant Wnt signaling and glycolytic metabolism.

## Conclusions

**Fig. 7:**
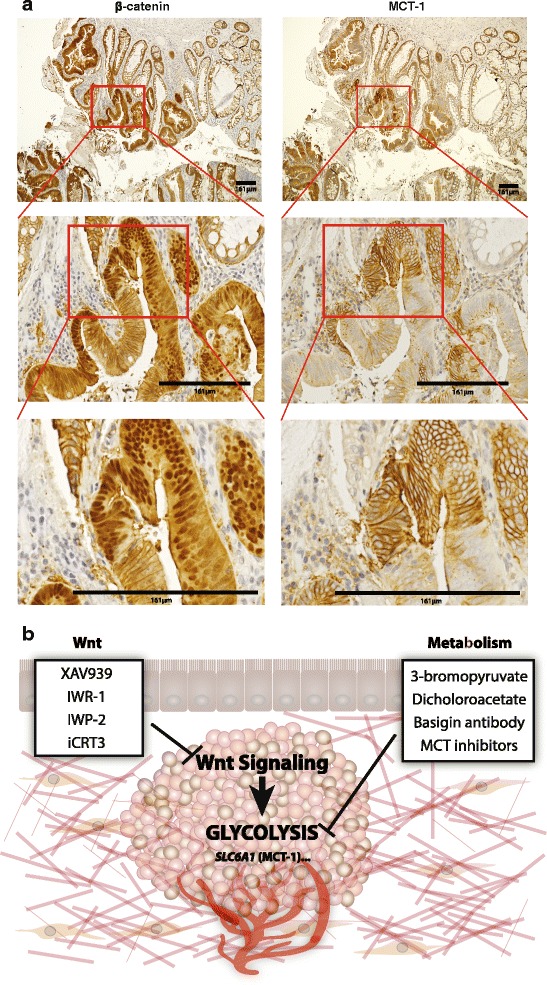
Wnt signaling influences cancer metabolism through regulation of *SLC16A1*/MCT-1 expression. **a** Immunohistochemical staining of human colon tumor samples (β-catenin and MCT-1) shows correlations between nuclear β-catenin and high levels of MCT-1. *Middle* and *bottom rows* show higher power images of *boxed* portions in the row above. **b** Model of a colorectal tumor with Wnt signaling regulating cancer metabolism through target genes including *SLC16A1*/MCT-1 expression. Examples of Wnt signaling and metabolic inhibitors currently under clinical development shown in *boxed* regions

Here, we report that *SLC16A1*/MCT-1 is a direct Wnt target gene within a program of glycolysis and angiogenesis that we have defined for colon cancer. We have previously shown that colon cancer cells with high levels of oncogenic Wnt signaling (Wnt^**Hi**^) have extremely strong signatures of aerobic glycolysis (Warburg metabolism) in vitro and in vivo [8]. MCT-1 upregulation in cells with oncogenic Wnt signaling supports their adoption of a glycolytic phenotype, likely through lactate efflux to maintain intracellular pH, but also through its unique ability to transport pyruvate which can influence the balance of glycolysis and oxidative phosphorylation [24]. Indeed, we observed that inhibition of β-catenin had marked effects on pyruvate transport (Fig. 2d). We find that colon cancer cells express one other monocarboxylate transporter (MCT-4) with high capacity for lactate, albeit with lower affinity. Mass spectrometry detection of intracellular and extracellular lactate indicates that lactate levels inside cells remain stable and low, even when MCT-1 expression is reduced (Additional file 2: Figure S2). We attribute the compensatory action to MCT-4 such that together, MCT-1 and MCT-4 provide sensitive and responsive maintenance of pH homeostasis for the intracellular environment. Current studies to examine the unique contributions of MCT-1 to the metabolic profile of colon cancer cells and tumors are ongoing. While previous studies have shown that c-Myc can activate *SLC16A1* transcription (as c-Myc:Max heterodimers), we find that this action works with LEF/TCF-β-catenin complexes in an additive manner. That is, the molecular actions of c-Myc:Max are not functionally dependent or synergistic with LEF/TCF-β-catenin. Since c-Myc is itself a Wnt target gene, *SLC16A1* transcription is targeted both indirectly (c-Myc) and directly (LEF/TCF-β-catenin complexes) by the oncogenic Wnt pathway, perhaps underscoring the importance of MCT-1 function in colon tumors. To further test for a correlation between Wnt signaling and MCT-1, we performed a limited case study of primary human colon tumors from four patients and used immunohistochemical staining to compare the expression patterns of β-catenin and MCT-1. We observed a striking correlation when there was obvious nuclear localization of β-catenin (Fig. 7a). However, we also note that MCT-1 expression exhibits a broad staining distribution in the epithelial portion of tumors (not in the stroma), but with heterogeneous patterns that reflect β-catenin staining localization (and therefore elevated Wnt signaling), as well as other patterns that do not necessarily correlate with β-catenin (see additional examples in Additional file 4: Figure S4). It is likely that additional signals and microenvironmental conditions exert important influence on *SLC16A1*/MCT-1 expression, influences that are also important to define now that we know MCT-1 is the single most important transporter of the candidate cancer drug 3-bromopyruvate.

Targeting metabolism is a promising avenue for treatment of colon cancer, and 3-bromopyruvate is a toxic small molecule that targets multiple enzymes in glycolysis, particularly in cells exhibiting high rates of Warburg metabolism [26, 39]. High levels of glycolysis result in the accumulation of metabolic products that alter intracellular pH, generating high levels of H^+^ that unless eliminated, would cause cell death. MCT proteins such as MCT-1 and MCT-4 can rid cancer cells of H^+^ via proton-coupled export of lactate, the “waste” end product of glycolysis, providing an important survival function for cancer cells. Also, MCT-1 is a pyruvate transporter, and as such can modulate the relative levels of glycolysis and oxidative phosphorylation. Thus, cells that have high levels of glycolytic activity tend to exhibit strong expression of MCT-1 and/or MCT-4 [40]. Studies with breast cancer cell lines show that 3-BP enters cells specifically and only through MCT-1. Signaling pathways and microenvironmental influences that regulate MCT-1 expression are therefore extremely important considerations for determining tumor sensitivities to this promising anti-cancer agent [35].

The discovery that Wnt signaling drives oncogenesis in many tumor types (including over 80 % of colorectal cancers) has inspired the development of small molecule inhibitors to target the pathway. Even though there is concern that Wnt signaling is necessary for stem cell compartments in normal tissues and therefore inhibitors will be deleterious and not tolerated, therapeutic windows have been demonstrated in animal experiments where Wnt-driven oncogenesis has been suppressed without general toxicity [41]. Thus, the therapeutic relevance of inhibiting the Wnt pathway in cancer remains, and multiple drugs that inhibit Wnt signaling are showing promise for clinical application (e.g., inhibitors of Wnt secretion, tankyrase 1/2 inhibitors, and inhibitors of β-catenin interactions) [42]. Here, we used XAV939 (a tankyrase inhibitor that promotes axin stabilization) to reduce Wnt signaling in the presence or absence of a metabolic inhibitor. XAV939, as well as a similar-acting compound called IWR-1, inhibits Wnt signaling downstream of ligand-receptor interaction at the level of the destruction complex. The destruction complex is a large, multi-subunit complex in the cytoplasm that targets β-catenin for degradation [43]. XAV939 treatment acts to stabilize the destruction complex and promote β-catenin degradation. Both XAV939 and IWR-1 are currently in pre-clinical development [42], though the low potency of these specific molecules has prompted the development of second generation drugs that target axin stabilization. For example, tankyrase inhibitors such as GM244-LM, a XAV939 analogue, and G007-LK are in the pipeline, both with greater specificity to tankyrase 1 and 2 [42, 44]. Interestingly, XAV939 increases sensitivity to chemotherapeutic drugs 5-fluorouracil and cisplatin in SW480 and SW620 cells, suggesting that Wnt inhibitors might be good candidates for combination with standard-of-care therapies [45], or possibly with new therapies that target metabolism such as 3-BP. Our work adds a note of caution to this latter idea as we show here that Wnt signaling is pivotal in upregulating the expression of MCT-1, the very transporter that 3-BP needs to gain access to cells.

Whether the level of Wnt signaling is the single most important indicator for cell sensitivity to 3-BP depends on understanding whether alternative modes exist for regulation of MCT-1 and whether the toxic activities of 3-BP target different processes inside cells. Our studies show that MCT-1 mRNA and protein levels largely correlated with Wnt signaling but that DLD-1 cells were an exception. DLD-1 cells are the most sensitive to 3-BP (IC_50_ = 23 μm), even though this cell line has low Wnt signaling. In fact, these cells have relatively high levels of MCT-1 mRNA and protein, similar to those in the Wnt^**Hi**^ SW480 cells (Fig. 2). There were also differences in the way the *SLC16A1* promoter responded to c-Myc and dnLEF-1 inhibition. The promoter had a slightly lower sensitivity to the c-Myc inhibitor (IC_50_ of 34 μM compared to SW480 cells (18 μM) and SW620 cells (10 μM)). To note, dnLEF-1 expression only made a noticeable difference at the lowest concentration of c-Myc inhibitor (Fig. 3), suggesting that there are relative differences in the way β-catenin/LEF and c-Myc contribute to promoter activity in DLD-1 cells. In general, c-Myc regulates two different kinetic steps of transcription at promoters: an early step of polymerase recruitment and initiation complex assembly, and pause-release at a later, downstream step [46]. Perhaps, the kinetics and contributions of these steps differ between DLD-1 and other cells. Whatever the mechanistic differences at the promoter, MCT-1 expression and by implication, 3-BP import potential, may have more to do with c-Myc than Wnt signaling in DLD-1 cells.

Finally, our data highlight interesting patterns of colon cancer cell growth when Wnt signaling inhibitors are combined with 3-BP treatment. Our hypothesis was that if MCT-1 is a target of Wnt signaling, and if MCT-1 transport is the mechanism by which 3-BP gains access to colon cancer cells, then inhibition of Wnt signaling should reduce sensitivity to 3-BP. We confirmed this hypothesis in the four cell lines, with the most obvious and notable effects emerging in SW480 and HCT116 cells. In these two cell lines, the IC_50_ for 3-BP inhibition was significantly shifted to higher concentrations when XAV939 was included in the cultures. Interestingly, even using concentrations of 3-BP that were so high as to reduce cell numbers to below the point of detection (300 μM), we observed that XAV939 co-treatment allowed cultures to recover better and faster after the drugs were washed out. It is particularly notable that even though XAV939 had only a small, albeit significant effect on protection in SW620 cells (Fig. 5b, d), its protective effects were more noticeable after the drug was removed (Fig. 6c). Meaning, even though there was no detectable live cells after 5 days of 3-BP treatment, XAV939-treated cultures retained a small number of viable cells which then recovered better and faster over the next 5 days. We speculate that XAV939 triggers adaptations that confer faster recovery and cell cycle progression. This possibility points to the caveats that can arise when two classes of drugs are combined. These data also suggest that the heterogeneous patterns of Wnt signaling that have been observed in primary human colon cancer, and within the tumor microenvironment, will directly affect the efficacy of 3-BP and its derivatives.

## Methods

### Cell lines/constructs

Colon cancer cell lines were grown under the following conditions: SW480 and SW620 were cultured in Dulbecco’s modified Eagle’s medium (DMEM; Fisher SH3008102) supplemented with 10 % fetal bovine serum (FBS; Atlas FP-0500-A) and 2 mM glutamine (Fisher MT-25-005-CI). HCT116 and DLD-1 were cultured in RPMI-1640 medium (Fisher MT15040CM) supplemented with 10 % FBS and 2 mM glutamine. Doxycycline-inducible DLD-1 cells were created by transfecting Tet-inducible dnLEF-1N into DLD-1 TR7 cells (a generous gift from M. van de Wetering and H. Clevers) as previously described [8]. The induction of dnLEF-1 was achieved through addition of 0.01 μg/ml doxycycline to the media. Lentiviral constructs were cloned via Cold Fusion (System Biosciences) by inserting the coding sequence for flag-tagged dnLEF-1N into pCDH lentivector (System Biosciences; SBI CD533A-2). See “Luciferase reporter plasmid cloning” section for details regarding TK and *SLC16A1* reporters.

### Lentiviral preparation and infection

Lentiviruses were prepared using System Biosciences lentivirus technology. Two-hundred ninety-three TN cells (System Biosciences (SBI) LV900A-1) were seeded in 150-mm plates at 7.5 × 10^6^ cells per plate with 20 mL DMEM without antibiotics for 24 h. Cells were then transfected with 22.5 μg pPACKH1 HIV packaging mix (SBI LV500A-1) and 4.5 μg of pCDH lentiviral vector using BioT transfection reagent. Viral supernatant was collected 48 and 72 h post-transfection. After centrifugation for 15 min at 3000×*g* to remove debris, 1× PEG-it (SBI LV810A-1) was added to precipitate virus. After incubation at 4 °C for at least 16 h, centrifugation (30 min at 1500×*g*) was used to collect viral particles. Virus was resuspended in a small volume (300–500 μL) 1× phosphate buffered saline (PBS) and titered using the Global UltraRapid Lentiviral Titer Kit (SBI LV961A-1). Transduction of target cells was performed according to manufacturer’s protocol (SBI). Briefly, cells were seeded at 1.0 × 10^5^ cells per 12-well or 2.5 × 10^5^ cells per six-well plate. After 24 h, cells were treated with fresh media, 1× TransDux (SBI LV850A-1), and lentivirus at a multiplicity of infection (MOI) of 10. MOI was determined using previously published methods. Infected cells were collected for subsequent assays after 72 h.

### Real-time PCR

Total RNA was isolated with Trizol from SW480 and SW620 expressing dnLEF-1. HCT116 cells were lentivirally transduced with dnLEF-1, and total RNA was isolated with Trizol 72 h post transduction. Total RNA was isolated with Trizol from DLD-1 cells after treatment with doxycycline for 72 h. A total of 2 μg of RNA were reverse transcribed using random primers according to the high capacity cDNA reverse transcription kit (Invitrogen 4374966). Real-time quantitative PCR (qRT-PCR) was performed with Maxima SYBR Green/ROX qPCR Master Mix (Fisher K0223). Relative change in gene expression was calculated using the ΔΔCt method using GAPDH expression for normalization. Statistical evaluation was performed by Student’s unpaired *t* test. *p* < 0.05 was considered statistically significant.

Primer pairs used for real-time PCR analysis include human *GAPDH* (5′-TCGACAGTCAGCCGCATCTTCTT-3′) and reverse (5′-GCGCCCAATACGACCAAATCC-3′), human *MCT-1* forward (5′-CACCGTACAGCAACTATACG-3′) and reverse (5′-CAATGGTCGCCTCTTGTAGA-3′), human *MCT-2* forward (5′-GGCTGGTTCCCTCATGAGAC-3′) and reverse (5′-GCTACCACAATAGCCCCAC-3′), human *MCT-3* forward (5′-TCGTGGGCTTCGTGGACAT-3′) and reverse (5′-GCACAACGCAGGCAGCAGTT-3′), human *MCT-4* forward (5′-ATTGGCCTGGTGCTGCTGATG-3′) and reverse (5′-CGAGTCTGCAGGAGGCTTGTG-3′).

### Western blot analysis

Cell lysates were prepared according to previously published methods [8] and 40 μg of lysate were analyzed by Western blot using the following antibodies: Lamin A/C (1:1000 Cell Signaling 2032), β-Tubulin (1:1000 GeneTex GTX107175), MCT-1 (1:1000 Santa Cruz SC-50324), β-catenin (1:1000 Cell Signaling #8480), and secondary antibody (1:5000 anti-rabbit IgG-horseradish peroxidase; Genesee 84-852). Blots were exposed to SuperSignal West Dura (Fisher PIA34075) and imaged on the Syngene GBox XL1.4 Imaging System.

### ^14^C Pyruvate uptake assay

SW480 cells were seeded at 4.0 × 10^4^ per 24-well 24 h prior to treatment. Cells were treated with 10 μM XAV939 or control DMSO in phenol-free DMEM. After 72 h of treatment, the ^14^C pyruvate-labeled uptake assay was performed at room temperature. Cells were first rinsed with room temperature HBSS (pH 6 with MES) and then incubated with 1 mM sodium pyruvate in HBSS with or without 0.5 μCi/mL ^14^C Pyruvate (Perkin Elmer NEC256050UC) for 0, 2, 15, 30, or 60 min. Following the incubation, the plates were placed on ice blocks to stop transport and rinsed with cold HBSS (pH 6 with MES). A cold stop solution of 2.2 mM HEPES and 0.21 M KCL was added to each well followed by cold 0.3 N NaOH with 0.1 % Triton-X. Plates were incubated at room temperature overnight prior to performing scintillation counts to collect CPM numbers for each sample. CPM numbers were normalized by protein via Bradford assay (Bio-Rad Quick Start 1× Bradford Dye 5000205). Statistical evaluation was performed by Student’s unpaired two tailed *t* test with *p* < 0.05 considered statistically significant.

### Luciferase reporter plasmid cloning

To create a luciferase reporter plasmid driven by the human *SLC16A1* or herpes virus thymidine kinase promoter, 5′ flanking sequences of the primers (see below; lower case) were designed for complementarity to the pGL2 or tkLUC vector backbone for use with a Cold Fusion cloning schema (Cold Fusion Cloning; System Biosciences). Human placental DNA was used as template for PCR amplification of the *SLC16A1 gene* core promoter using Pfu Turbo polymerase. The PCR fragment and SmaI-digested pGL2 vector was purified following the manufacturer’s protocol (GeneJET Gel Extraction Kit, Fisher K0691), and ligations were performed with an insert/plasmid ratio of 1:2 with Cold Fusion reagents. Clones were verified by sequencing. The following PCR primers were used:

Forward primer (−1604), upper case sequence is genomic, and lower case sequence is complementary to pGL2): 5′-gag cta aca taa ccc TCC TGG GAT TCA TCT TAT TT-3′

Reverse primer (+1045), upper case sequence is genomic, and lower case sequence is complementary to pGL2): 5′-agc tcg gta cct ccc tAT CCT CCA GAT TTC TCT CA-3′

The following PCR primer sequences were used to amplify a region identified as occupied by TCF-1, for cloning 5′ of the heterologous herpes virus tk promoter (tkLUC):

Primers designed for amplification and insertion into a plasmid backbone containing a minimal Herpes Virus thymidine kinase reporter (at BamHI):

ChIP Peak (486 nt; chromosome 1 113499604-113500089)

Forward primer (upper case sequence is genomic, and lower case is complementary to tkLUC):

5′-atc tta tca tgt ctg TCC TGG CAA GCA GCA-3′

Reverse primer (upper case sequence is genomic, and lower case is complementary to tkLUC):

5′-ctc gga ccc cgg atc GTG GGT TGG GGT GTG-3′

### Luciferase assay

Cells were seeded at 2.5 × 10^5^ per six-well 24 h prior to transfection. Each well was transfected with 0.5 μg M50 Super 8×TOPflash (a gift from Dr. RT Moon; Addgene plasmid 12456), TK, or *SLC16A1* luciferase reporters and 0.1 μg thymidine kinase β-galactosidase plasmid using BioT transfection reagent (Bioland Scientific B01-02). Cells were transfected with 0.01 μg/mL dnLEF-1 and/or treated with 10 μM XAV939 at the time of reporter transfection where indicated. Cells were harvested 24-h post transfection and assayed for luciferase activity and β-galactosidase activity (used for normalization). Statistical evaluation was performed by Student’s unpaired *t* test. *p* < 0.05 was considered statistically significant.

### Sulforhodamine B (SRB) cell growth assay

For 3-bromopyruvate dose-response curves, SW480 and SW620 cells were seeded in 96-well plates at 5000 cells per well with eight replicates for each condition. HCT116 and DLD-1 cells were seeded in 96-well plates at 2500 cells per well, optimized to obtain confluent cultures around day 7 of each experiment, with eight replicates for each condition. Twenty-four hours after seeding, cells were pre-treated with 10 μM XAV939 for 48 h, followed by 3-BPr for a period of 96 h. Cells were then fixed 96 h after 3-BPr treatment and stained with sulforhodamine B according to published protocols [47] with optical density readings performed at 492 nm.

For drug wash-out survival assays, cells were seeded in 96-well plates at 5000 cells per well with eight replicates for each condition. Twenty-four hours after seeding, cells were treated for a period of 96 h (4 days). On day 5, media containing treatment was removed and replaced with media containing no treatment to begin “recovery” period. Cells were fixed on days 5–10, with days 0–5 considered post wash-out, and stained according to published protocols [47]. Optical density readings were performed at 492 nm.

Statistical evaluation was performed by Student’s unpaired *t* test. *p* < 0.05 was considered statistically significant.

### Immunohistochemistry

For MCT-1 and β-catenin staining in human colon tumor samples, following pressure cooker antigen retrieval in citrate buffer, adjacent sections were blocked in 3 % H_2_O_2_, goat serum, and avidin-biotin blocking reagent (Vector Labs). Sections were incubated with primary antibody solutions: anti-MCT-1 (Santa Cruz SC-50324, 1:500) or anti-β-catenin (BD Biosciences 610154, 1:500), followed by biotinylated secondary antibodies and visualization using a peroxidase-conjugated avidin-based Vectastain protocol. Slides were then counterstained with hematoxylin and mounted.

## Additional files

Additional file 1: Figure S1. Blocking Wnt with XAV939 reduces MCT-1 levels. qRT-PCR analysis was performed on RNA collected from SW480 (A), SW620 (B) HCT116 (C) and DLD-1 (D) cells treated with XAV939 (10 μM) for 24 h. Graphs shown represent the average of three trials (+/− SEM). Whole cell lysates from each cell line (A-D) were harvested 72 h (for MCT-1) and 24 h (for β-catenin) after XAV939 treatment (10 μM) and were probed with the antibodies shown. (**p* value < 0.05; ***p* value < 0.01; ****p* value < 0.001). (PDF 678 kb) Additional file 2: Figure S2. Disruption of oncogenic signaling leads to decreased lactate production and increased GSSG levels. SW480 cell cultures treated with vehicle (DMSO) or XAV inhibitor, or established cell lines transfected with either empty vector (mock) or a dnLEF-1 construct, were grown to 80 % confluency in 10-cm tissue culture plates in 10 % FBS supplemented DMEM medium. Cells and conditioned media were collected for metabolite extraction as follows. Briefly, cells were harvested from plates by trypsinization, counted and 4–5 replicate aliquots of 10e6 cells/sample prepared. Cells or media were collected by centrifugation at 1200×*g* for 5 min, rinsed with phosphate buffered saline, and extracted with 75 % ethanol/10 mM HEPES pH 7.4 buffer (final) at 80 °C for 5 min. Lysates were cleared by centrifugation at 12,000×*g* for 10 min at 4 °C. Supernatants were lyophilized, resuspended in 10 mM ammonium formate buffer, and cleared supernatant transferred to low volume mass spectrometry sample vials. Panel (A) lactic acid and panel (B) oxidized glutathione (GSSG) were quantitated by UPLC ESI MSMS on a Waters Quattro Premier XE instrument using ESI− and ESI+ ion modes, respectively. Analyte concentrations of lactic acid and GSSG were calculated from 8-point calibration standard curves in the range of 0.1–300 μM, quadratic fit, *r* ^2^ > 0.98. Data are shown for intracellular and media concentrations as scatter plots with means ± SEM, *n* = 4–5. ***p* < 0.01, ****p* < 0.001. Instrument settings for lactic acid: UPLC gradient—solvent A: water + 0.1 % formic acid, solvent B: 50 % acetonitrile: 50 % isopropanol + 0.1 % formic acid. Initial 10 % B→90 % B in 3 min, hold 90 % B for 1 min. MS tuning settings: SRM 89→47, CV 20, CE 50, RT 0.45 min. Instrument settings for GSSG: UPLC gradient—solvent A: water + 0.2 % acetic acid, B acetonitrile + 0.2 % acetic acid. Initial 10 % B→90 % B in 3 min, hold 1 min. MS tuning settings, SRM 613→231, CV 20, CE 30, RT 0.58 min. Column: Waters UPLC C18 BEH column, 1.7 μM, 2.1 × 50 mm. (PDF 305 kb) Additional file 3: Figure S3.
*SLC16A1* transcription is directly regulated by LEF/TCFs and Wnt signaling. (A) Schematic of one regulatory region located approximately 624 nt upstream from the *SLC16A1* transcription start site (+1) is occupied by dnTCF-1 and contains two putative Wnt response elements (WREs). Genomic location and sequence show putative WREs highlighted in red. (B) SuperTopflash reporter serves as a positive control for luciferase activity assays in parental SW480, SW620, HCT116, and DLD-1 cells. The SuperTopflash reporter is significantly sensitive to repression by dnLEF-1. (PDF 318 kb) Additional file 4: Figure S4. MCT-1 and β-catenin staining in human colon tumor samples. Immunohistochemical staining of four human colon tumor samples (β-catenin and MCT-1) shows heterogeneous patterns for both β-catenin and MCT-1. β-catenin and MCT-1 were stained in adjacent tumor slices. Images shown at ×20 magnification. (PDF 31 kb)

## Abbreviations

3-BP: 3-Bromopyruvate
AICAR: Aminoimidazole-4-carboxamide-1-β-d-ribonucleoside
AMPK: AMP-activated protein kinase
dnLEF/TCFs: Dominant-negative LEF/TCF transcription factors
dnLEF-1: Dominant-negative LEF-1
EMT: Epithelial-mesenchymal transition
GSSG: Glutathione disulfide
MCT-1: Monocarboxylate transporter-1
NFAT: Nuclear factor of activated T-cells
PDK1: Pyruvate dehydrogenase kinase 1
SRB: Sulforhodamine B
WREs: Wnt response elements

## References

[1] Reya T, Clevers H (2005). Wnt signalling in stem cells and cancer. Nature.

[2] Bienz M, Clevers H (2000). Linking colorectal cancer to Wnt signaling review. Cell.

[3] Klaus A, Birchmeier W (2008). Wnt signalling and its impact on development and cancer. Nat Rev Cancer.

[4] Clevers H (2006). Wnt/beta-catenin signaling in development and disease. Cell.

[5] Van de Wetering M (2002). The β-catenin/TCF-4 complex imposes a crypt progenitor phenotype on colorectal cancer cells. Cell.

[6] Hoverter NP, Ting J-H, Sundaresh S, Baldi P, Waterman ML (2012). A WNT/p21 circuit directed by the C-clamp, a sequence-specific DNA binding domain in TCFs. Mol Cell Biol.

[7] Brabletz T (2005). Invasion and metastasis in colorectal cancer: epithelial-mesenchymal transition, mesenchymal-epithelial transition, stem cells and beta-catenin. Cells Tissues Organs.

[8] Pate KT (2014). Wnt signaling directs a metabolic program of glycolysis and angiogenesis in colon cancer. EMBO J.

[9] Schuijers J, Mokry M, Hatzis P, Cuppen E, Clevers H (2014). Wnt-induced transcriptional activation is exclusively mediated by TCF/LEF. EMBO J.

[10] Batlle E (2002). β-catenin and TCF mediate cell positioning in the intestinal epithelium by controlling the expression of EphB/EphrinB. Cell.

[11] Izumi H (2011). Monocarboxylate transporters 1 and 4 are involved in the invasion activity of human lung cancer cells. Cancer Sci.

[12] Pinheiro C (2012). Role of monocarboxylate transporters in human cancers: state of the art. J Bioenerg Biomembr.

[13] Halestrap AP (2013). Monocarboxylic acid transport. Compr Physiol.

[14] Halestrap AP, Wilson MC (2012). The monocarboxylate transporter family—role and regulation. IUBMB Life.

[15] Majumdar S, Gunda S, Pal D, Mitra AK (2005). Functional activity of a monocarboxylate transporter, MCT1, in the human retinal pigmented epithelium cell line, ARPE-19. Mol Pharm.

[16] Benton CR (2008). PGC-1alpha increases skeletal muscle lactate uptake by increasing the expression of MCT1 but not MCT2 or MCT4. Physiol Genomics.

[17] Galardo MN, Riera MF, Pellizzari EH, Cigorraga SB, Meroni SB (2007). The AMP-activated protein kinase activator, 5-aminoimidazole-4-carboxamide-1-b-D-ribonucleoside, regulates lactate production in rat Sertoli cells. J Mol Endocrinol.

[18] Cuff MA, Lambert DW, Shirazi-Beechey SP (2002). Substrate-induced regulation of the human colonic monocarboxylate transporter, MCT1. J Physiol.

[19] Perez de Heredia F, Wood IS, Trayhurn P (2010). Hypoxia stimulates lactate release and modulates monocarboxylate transporter (MCT1, MCT2, and MCT4) expression in human adipocytes. Pflugers Arch.

[20] Ullah MS, Davies AJ, Halestrap AP (2006). The plasma membrane lactate transporter MCT4, but not MCT1, is up-regulated by hypoxia through a HIF-1alpha-dependent mechanism. J Biol Chem.

[21] Boidot R (2012). Regulation of monocarboxylate transporter MCT1 expression by p53 mediates inward and outward lactate fluxes in tumors. Cancer Res.

[22] Doherty JR (2014). Blocking lactate export by inhibiting the Myc target MCT1 disables glycolysis and glutathione synthesis. Cancer Res.

[23] Dhup S, Dadhich RK, Porporato PE, Sonveaux P (2012). Multiple biological activities of lactic acid in cancer: influences on tumor growth, angiogenesis and metastasis. Curr Pharm Des.

[24] Hong CS (2016). MCT1 modulates cancer cell pyruvate export and growth of tumors that co-express MCT1 and MCT4. Cell Rep.

[25] Le Floch R (2011). CD147 subunit of lactate/H+ symporters MCT1 and hypoxia-inducible MCT4 is critical for energetics and growth of glycolytic tumors. Proc Natl Acad Sci U S A.

[26] Birsoy K (2013). MCT1-mediated transport of a toxic molecule is an effective strategy for targeting glycolytic tumors. Nat Genet.

[27] El Sayed SM (2014). Safety and outcome of treatment of metastatic melanoma using 3-bromopyruvate: a concise literature review and case study. Chin J Cancer.

[28] Ko YH (2012). A translational study “case report” on the small molecule “energy blocker” 3-bromopyruvate (3BP) as a potent anticancer agent: from bench side to bedside. J Bioenerg Biomembr.

[29] Watanabe K (2014). Integrative ChIP-seq/microarray analysis identifies a CTNNB1 target signature enriched in intestinal stem cells and colon cancer. PLoS One.

[30] Huang S-MA (2009). Tankyrase inhibition stabilizes axin and antagonizes Wnt signalling. Nature.

[31] Veeman MT, Slusarski DC, Kaykas A, Louie SH, Moon RT (2003). Zebrafish prickle, a modulator of noncanonical Wnt/Fz signaling, regulates gastrulation movements. Curr Biol.

[32] Chiche J (2012). In vivo pH in metabolic-defective Ras-transformed fibroblast tumors: key role of the monocarboxylate transporter, MCT4, for inducing an alkaline intracellular pH. Int J Cancer.

[33] Hoverter NP (2014). The TCF C-clamp DNA binding domain expands the Wnt transcriptome via alternative target recognition. Nucleic Acids Res.

[34] Baltazar F (2014). Monocarboxylate transporters as targets and mediators in cancer therapy response. Histol Histopathol.

[35] Morais-Santos F (2015). Targeting lactate transport suppresses in vivo breast tumour growth. Oncotarget.

[36] Sonveaux P (2008). Targeting lactate-fueled respiration selectively kills hypoxic tumor cells in mice. J Clin Invest.

[37] Baba M, Inoue M, Itoh K, Nishizawa Y (2008). Blocking CD147 induces cell death in cancer cells through impairment of glycolytic energy metabolism. Biochem Biophys Res Commun.

[38] Kirk P (2000). CD147 is tightly associated with lactate transporters MCT1 and MCT4 and facilitates their cell surface expression. EMBO J.

[39] Nilsson H (2015). Primary clear cell renal carcinoma cells display minimal mitochondrial respiratory capacity resulting in pronounced sensitivity to glycolytic inhibition by 3-Bromopyruvate. Cell Death Dis.

[40] Parks SK, Chiche J, Pouyssegur J (2011). pH control mechanisms of tumor survival and growth. J Cell Physiol.

[41] Proffitt KD (2013). Pharmacological inhibition of the Wnt acyltransferase PORCN prevents growth of WNT-driven mammary cancer. Cancer Res.

[42] Kahn M (2014). Can we safely target the WNT pathway?. Nat Rev Drug Discov.

[43] Stamos JL, Weis WI. The β-catenin destruction complex. Cold Spring Harb Perspect Biol. 2013;5. 10.1101/cshperspect.a007898 PMC3579403 23169527

[44] Lau T (2013). A novel tankyrase small-molecule inhibitor suppresses APC mutation-driven colorectal tumor growth. Cancer Res.

[45] Wu X, Luo F, Li J, Zhong X, Liu K. Tankyrase 1 inhibitior XAV939 increases chemosensitivity in colon cancer cell lines via inhibition of the Wnt signaling pathway. Int J Oncol. 2013;5(1):a007898. 10.3892/ijo.2016.3360 PMC4777596 26820603

[46] Rahl PB (2010). C-Myc regulates transcriptional pause release. Cell.

[47] Monici M (2003). Dependence of leukemic cell autofluorescence patterns on the degree of differentiation. Photochem Photobiol Sci.

