# Supplementary figures and images for: Lactate/pyruvate transporter MCT-1 is a direct Wnt target that confers sensitivity to 3-bromopyruvate in colon cancer
Source: Cancer Metab. 2016 Oct 3;4:20. doi: 10.1186/s40170-016-0159-3 (PMC5046889; doi:10.1186/s40170-016-0159-3)

Figure S1

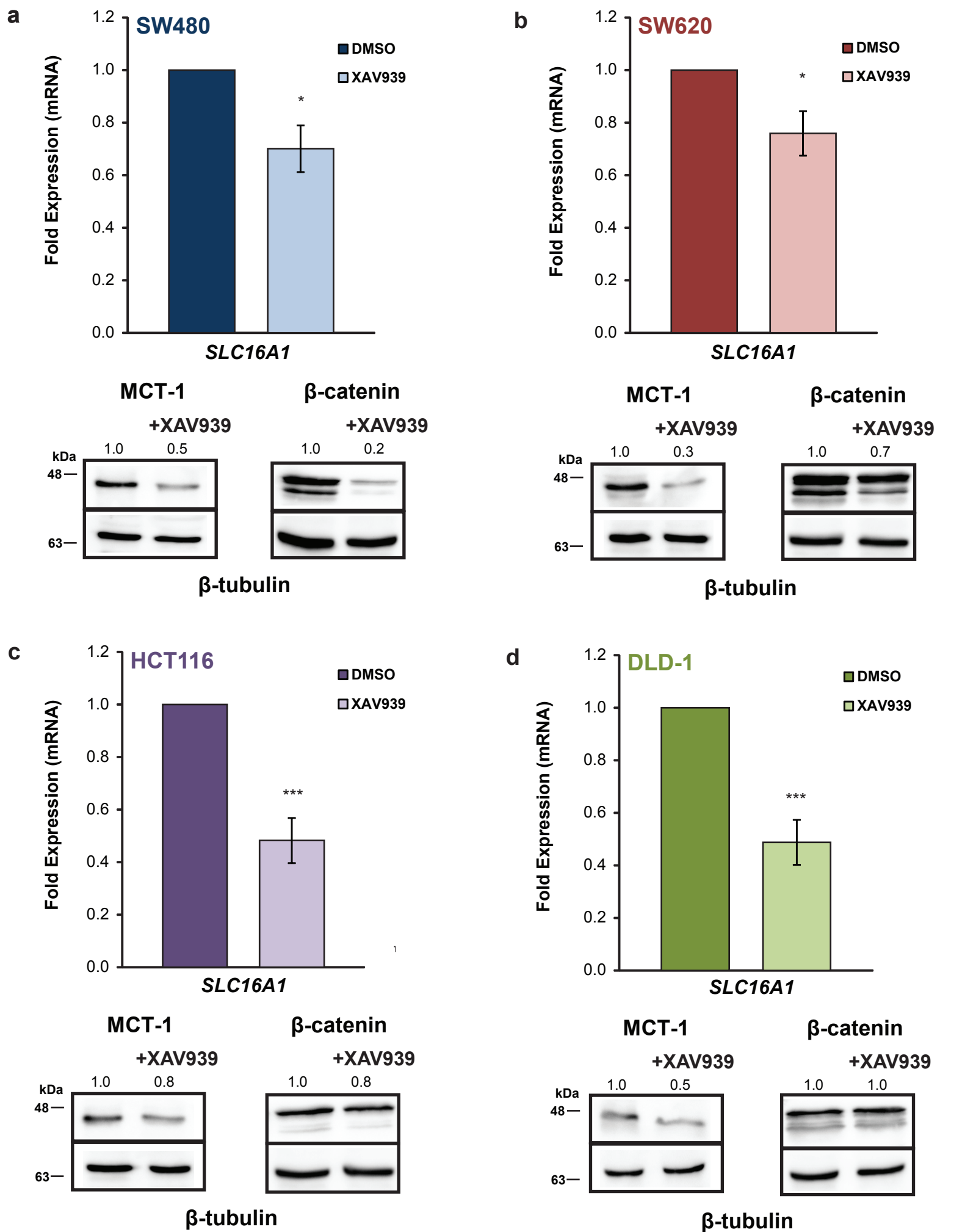

Supplement: Additional file 1: Figure S1. — Blocking Wnt with XAV939 reduces MCT-1 levels. qRT-PCR analysis was performed on RNA collected from SW480 (A), SW620 (B) HCT116 (C) and DLD-1 (D) cells treated with XAV939 (10 μM) for 24 h. Graphs shown represent the average of three trials (+/− SEM). Whole cell lysates from each cell line (A-D) were harvested 72 h (for MCT-1) and 24 h (for β-catenin) after XAV939 treatment (10 μM) and were probed with the antibodies shown. (*p value < 0.05; **p value < 0.01; ***p value < 0.001). (PDF 678 kb) [file 40170_2016_159_MOESM1_ESM.pdf]

Figure S2

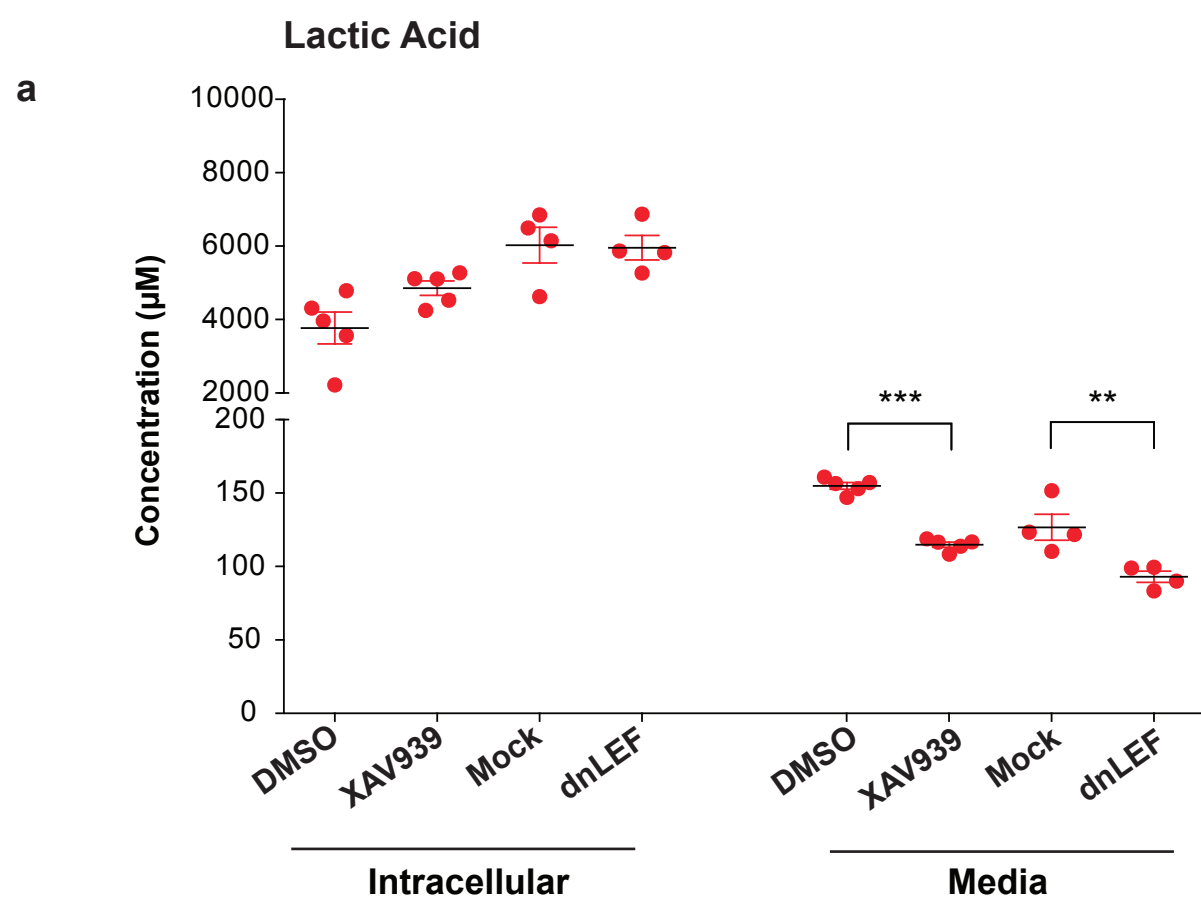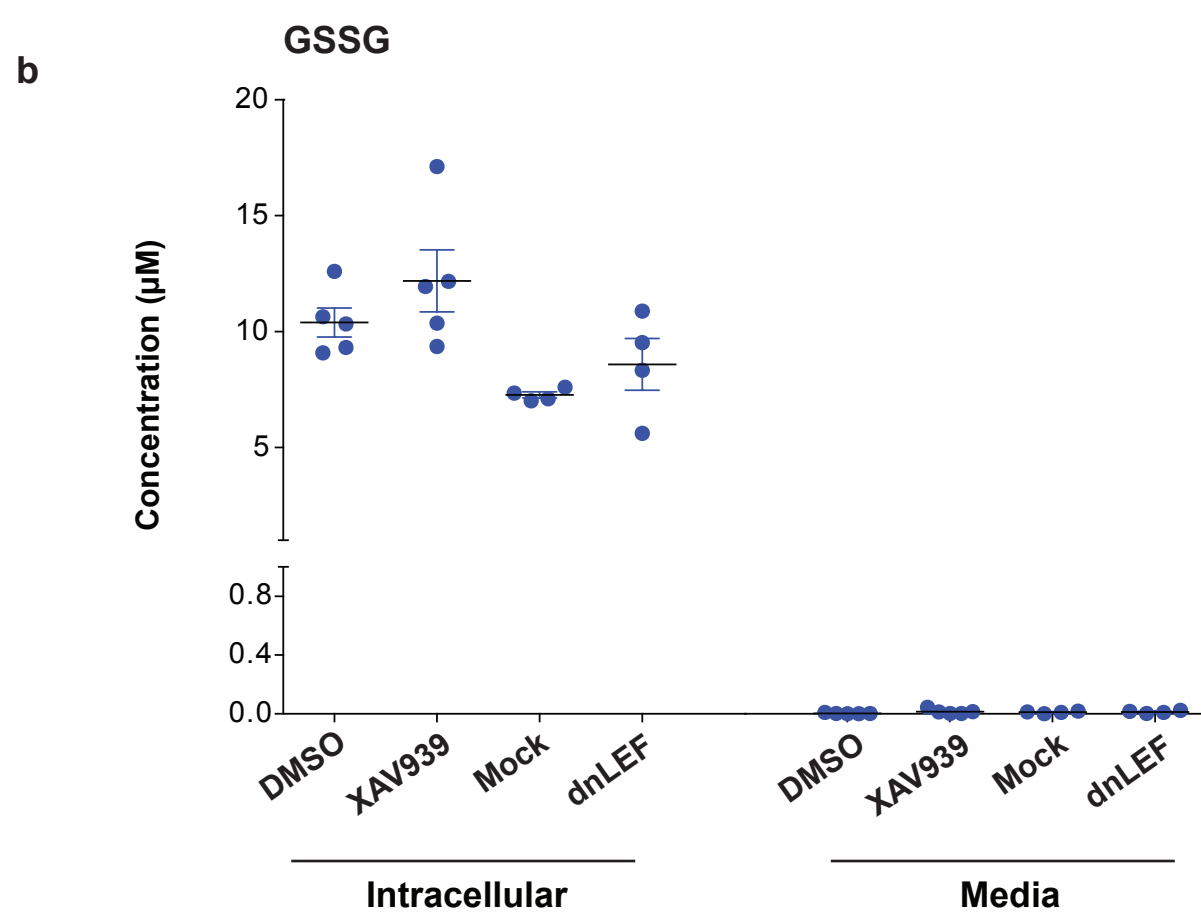

Supplement: Additional file 2: Figure S2. — Disruption of oncogenic signaling leads to decreased lactate production and increased GSSG levels. SW480 cell cultures treated with vehicle (DMSO) or XAV inhibitor, or established cell lines transfected with either empty vector (mock) or a dnLEF-1 construct, were grown to 80 % confluency in 10-cm tissue culture plates in 10 % FBS supplemented DMEM medium. Cells and conditioned media were collected for metabolite extraction as follows. Briefly, cells were harvested from plates by trypsinization, counted and 4–5 replicate aliquots of 10e6 cells/sample prepared. Cells or media were collected by centrifugation at 1200×g for 5 min, rinsed with phosphate buffered saline, and extracted with 75 % ethanol/10 mM HEPES pH 7.4 buffer (final) at 80 °C for 5 min. Lysates were cleared by centrifugation at 12,000×g for 10 min at 4 °C. Supernatants were lyophilized, resuspended in 10 mM ammonium formate buffer, and cleared supernatant transferred to low volume mass spectrometry sample vials. Panel (A) lactic acid and panel (B) oxidized glutathione (GSSG) were quantitated by UPLC ESI MSMS on a Waters Quattro Premier XE instrument using ESI− and ESI+ ion modes, respectively. Analyte concentrations of lactic acid and GSSG were calculated from 8-point calibration standard curves in the range of 0.1–300 μM, quadratic fit, r 2 > 0.98. Data are shown for intracellular and media concentrations as scatter plots with means ± SEM, n = 4–5. **p < 0.01, ***p < 0.001. Instrument settings for lactic acid: UPLC gradient—solvent A: water + 0.1 % formic acid, solvent B: 50 % acetonitrile: 50 % isopropanol + 0.1 % formic acid. Initial 10 % B→90 % B in 3 min, hold 90 % B for 1 min. MS tuning settings: SRM 89→47, CV 20, CE 50, RT 0.45 min. Instrument settings for GSSG: UPLC gradient—solvent A: water + 0.2 % acetic acid, B acetonitrile + 0.2 % acetic acid. Initial 10 % B→90 % B in 3 min, hold 1 min. MS tuning settings, SRM 613→231, CV 20, CE 30, RT 0.58 min. Column: Waters UPLC C18 BEH column, 1.7 μM, 2. [file 40170_2016_159_MOESM2_ESM.pdf]

**Figure S4**

**$\beta$ -catenin**

**MCT-1**

**Tumor #1**

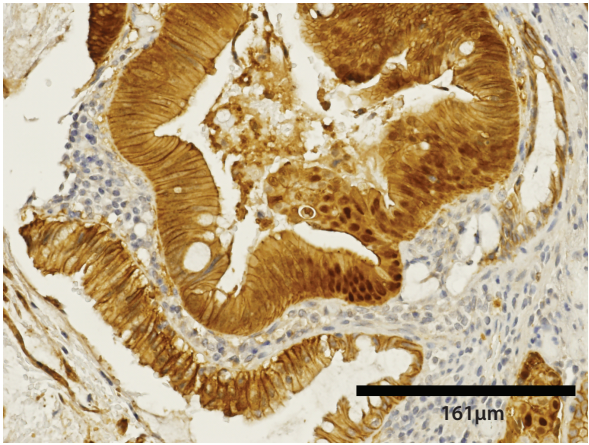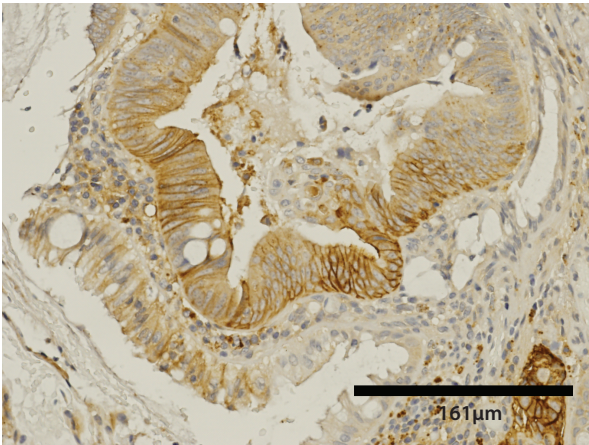

**Tumor #2**

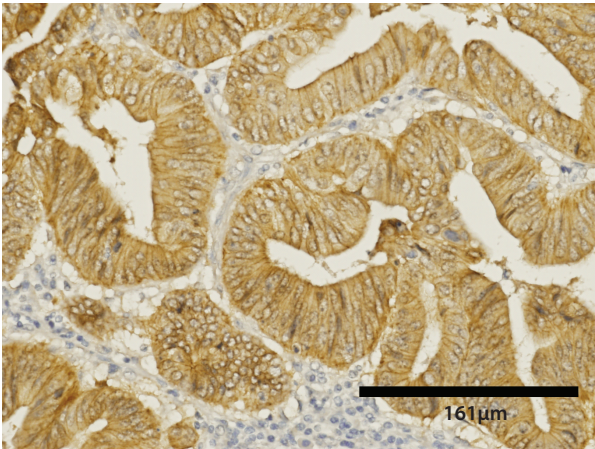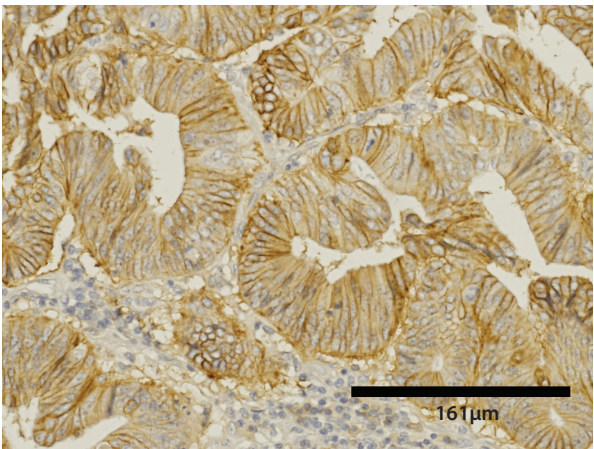

**Tumor #3**

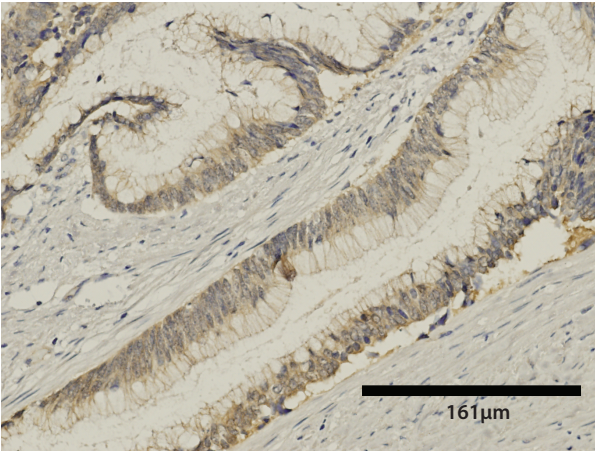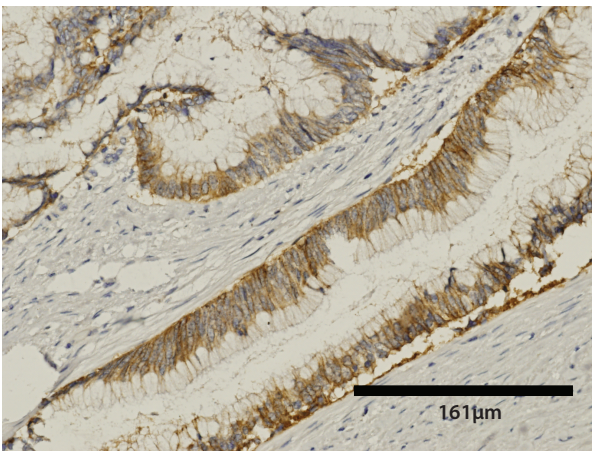

**Tumor #4**

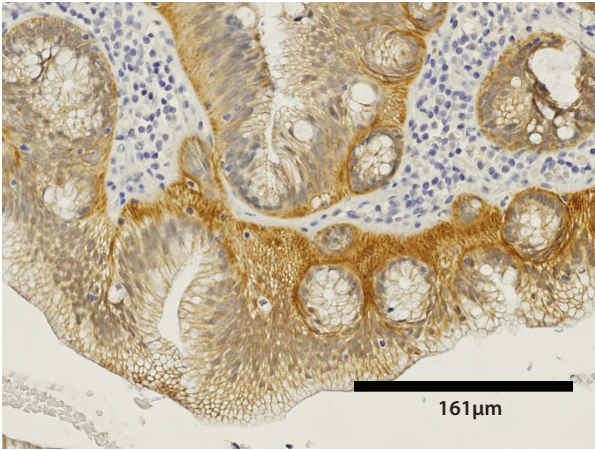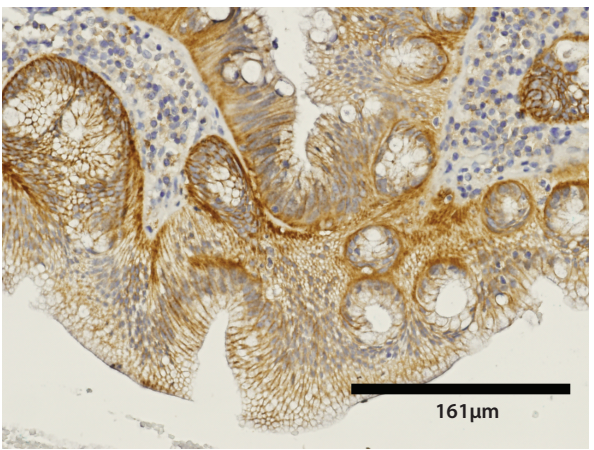

Supplement: Additional file 4: Figure S4. — MCT-1 and β-catenin staining in human colon tumor samples. Immunohistochemical staining of four human colon tumor samples (β-catenin and MCT-1) shows heterogeneous patterns for both β-catenin and MCT-1. β-catenin and MCT-1 were stained in adjacent tumor slices. Images shown at ×20 magnification. (PDF 31 kb) [file 40170_2016_159_MOESM4_ESM.pdf]
