# Supplementary material for: Lactate/pyruvate transporter MCT-1 is a direct Wnt target that confers sensitivity to 3-bromopyruvate in colon cancer
Source: Cancer Metab. 2016 Oct 3;4:20. doi: 10.1186/s40170-016-0159-3 (PMC5046889; doi:10.1186/s40170-016-0159-3)

Figure S3

a

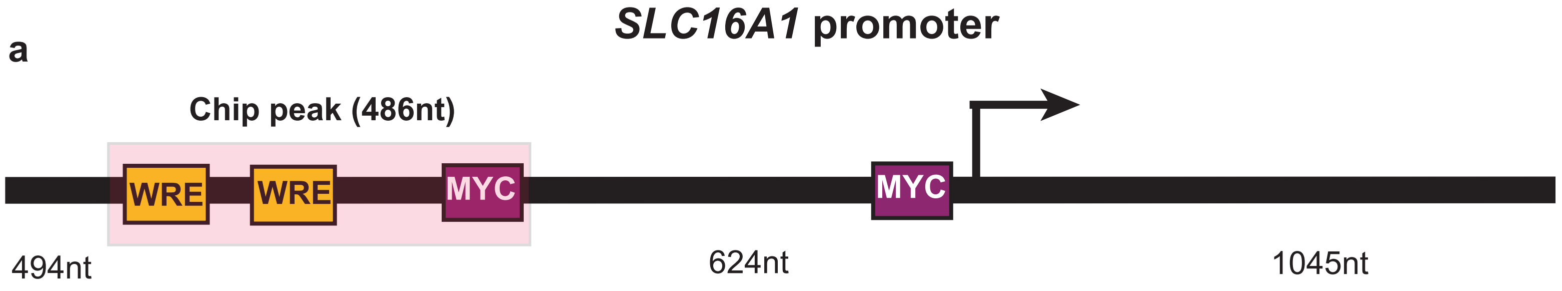

Chip peak: Chr1 113499604-113500089

TCCTGGCAAGCAGCAAGTGCTCAACAGATGTTGGTTAATTTGTGCCTAAGTCTCCCCCAAAAACTTGCAAGCT
CTTTGAAGGCAGGGGCCTTGCCTCATATTTTTCGGTATTTCTCAAAAGTCCTGGACATCAAAGACACTCCATGG
TTATTTGATCACTTCAACACACACGTTGGTTATGCGGTCACAGCGGCTATTATGTTAAAAACAGGCTCTGGAGT
GTGACTTCAAATGCTGGCCGCTGACGAGCTGGGCTGTCTGGAAAGTTACTTCACCTCTCTGAGCCTCGGGTC
CCTTATTTACAAAATGTTCAGGGCAGTGCCCACTTCACAGGGCTGAGGATTAGAGAGGATAATCCGGGTGAGG
AGCATCAGCCCCAGCGCCGCCTGCCACAATTCTATCCGGCCCCACATATGCATCGTCCATAAGCGTCCGGCCT
CTGCATTCTCGCTTTTCCACGTGGCCGAGGTGCACACCCCAACCCAC

b

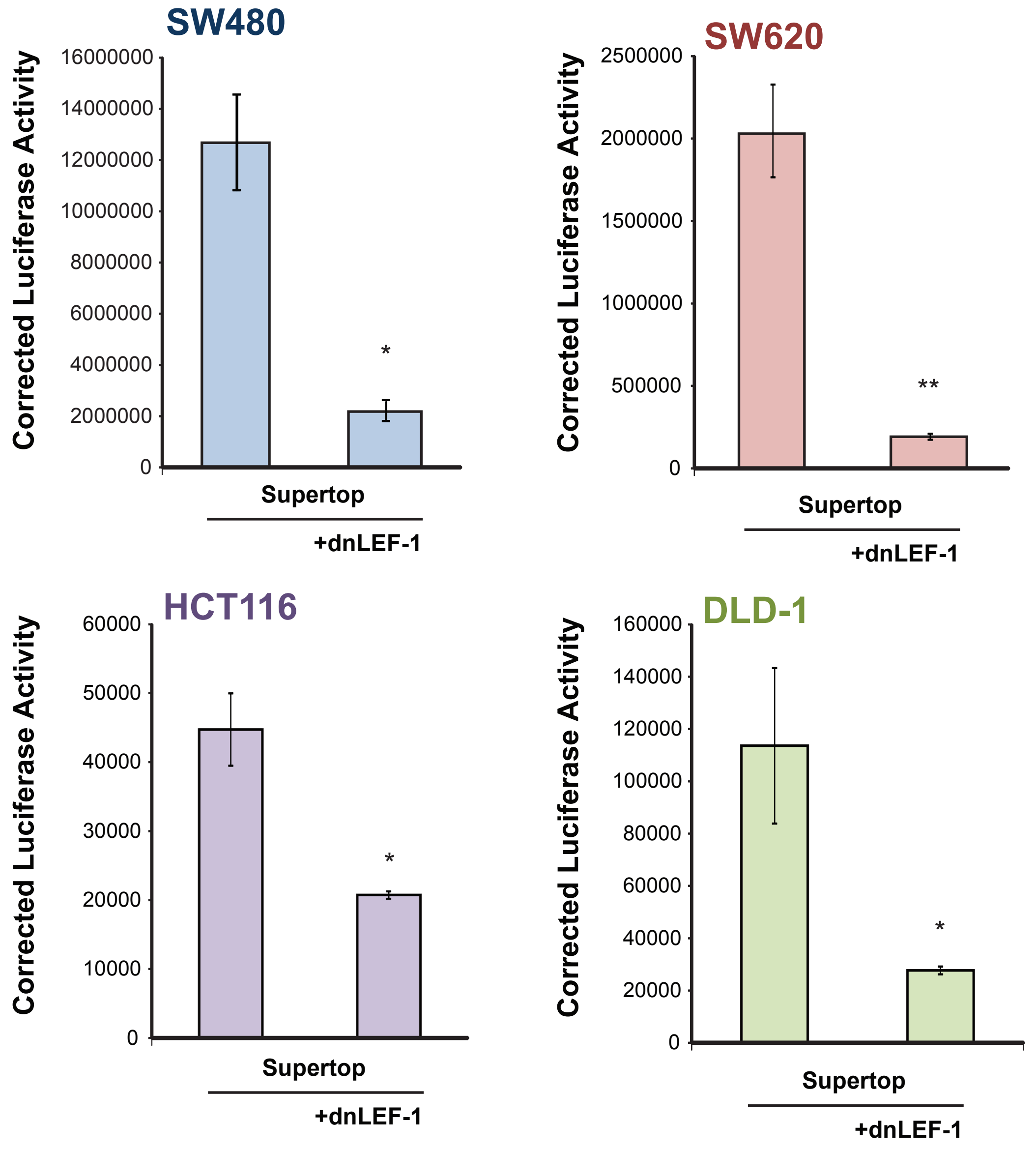

Supplement: Additional file 3: Figure S3. — SLC16A1 transcription is directly regulated by LEF/TCFs and Wnt signaling. (A) Schematic of one regulatory region located approximately 624 nt upstream from the SLC16A1 transcription start site (+1) is occupied by dnTCF-1 and contains two putative Wnt response elements (WREs). Genomic location and sequence show putative WREs highlighted in red. (B) SuperTopflash reporter serves as a positive control for luciferase activity assays in parental SW480, SW620, HCT116, and DLD-1 cells. The SuperTopflash reporter is significantly sensitive to repression by dnLEF-1. (PDF 318 kb) [file 40170_2016_159_MOESM3_ESM.pdf]
